# Full UPF3B function is critical for neuronal differentiation of neural stem cells

**DOI:** 10.1186/s13041-015-0122-1

**Published:** 2015-05-27

**Authors:** Tahani Alrahbeni, Francesca Sartor, Jihan Anderson, Zosia Miedzybrodzka, Colin McCaig, Berndt Müller

**Affiliations:** University of Aberdeen, Institute of Medical Sciences, Foresterhill, Aberdeen AB25 2ZD, Scotland, UK; Current address: Riyadh Colleges of Dentistry and Pharmacy, Olaya Campus, Riyadh, Saudi Arabia; Medical Genetics, Polwarth Building, Foresterhill, Aberdeen AB25 2ZD, Scotland, UK

**Keywords:** Autism, Schizophrenia, X-linked intellectual disability, Nonsense-mediated mRNA decay, *UPF3B* mutation, *UPF1*, Tethered function assay, qPCR, Arhgap24, Atf4, Protein localisation

## Abstract

**Background:**

Mutation in the *UPF3B* gene on chromosome X is implicated in neurodevelopmental disorders including X-linked intellectual disability, autism and schizophrenia. The protein UPF3B is involved in the nonsense-mediated mRNA decay pathway (NMD) that controls mRNA stability and functions in the prevention of the synthesis of truncated proteins.

**Results:**

Here we show that NMD pathway components UPF3B and UPF1 are down-regulated during differentiation of neural stem cells into neurons. Using tethered function assays we found that *UPF3B* missense mutations described in families with neurodevelopmental disorders reduced the activity of UPF3B protein in NMD. In neural stem cells, UPF3B protein was detected in the cytoplasm and in the nucleus. Similarly in neurons, UPF3B protein was detected in neurites, the somatic cytoplasm and in the nucleus. In both cell types nuclear UPF3B protein was enriched in the nucleolus. Using GFP tagged UPF3B proteins we found that the missense mutations did not affect the cellular localisation. Expression of missense mutant UPF3B disturbed neuronal differentiation and reduced the complexity of the branching of neurites. Neuronal differentiation was similarly affected in the presence of the NMD inhibitor Amlexanox. The expression of mutant UPF3B proteins lead to a subtle increase in mRNA levels of selected NMD targets.

**Conclusions:**

Together our findings indicate that, despite the down-regulation of NMD factors, functional NMD is critical for neuronal differentiation. We propose that the neurodevelopmental phenotype of UPF3B missense mutation is caused by impairment of NMD function altering neuronal differentiation.

**Electronic supplementary material:**

The online version of this article (doi:10.1186/s13041-015-0122-1) contains supplementary material, which is available to authorized users.

## Background

**Fig. 1:**
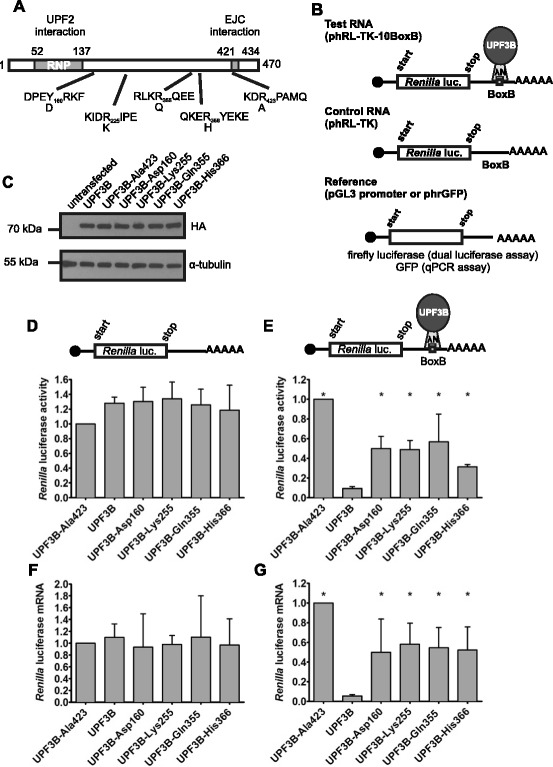
Missense mutations impair UPF3B activity in NMD. **a**. Schematic representation of UPF3B protein structure with location of protein-protein interaction domains and amino acid changes. N-terminal N peptide and HA tag sequences are not shown. **b**. Schematic representation of the tethering assay mimicking NMD. λNUPF3B protein is tethered to *Renilla* luciferase mRNA with BoxB elements in the 3’ UTR expressed from phRL-TK-10BoxB (Test RNA) but is unable to bind mRNA expressed from phRL-TK lacking BoxB elements (Control RNA). Co-transfected pGL3-promoter or phrGFP serve as standards in dual luciferase and qPCR assays, respectively (Reference). **c**. Expression of λN-HA-UPF3B proteins. HeLa cells were transfected with pCI-λN-HA-UPF3B expression constructs and lysed after 48 h. Expression of UPF3B proteins and α-tubulin was analysed by 10 % SDS PAGE followed by Western blotting with anti-HA and anti-tubulin antibodies. **d**, **e**. Tethering assay: Luciferase activity. Hela cells were transfected with phRL-TK (**d**) or phRL-TK-10BoxB (**e**), together with reference plasmid pGL3-promoter and the pCI-λN-HA-UPF3B expression constructs. Luciferase activities were measured 48 h after transfection. *Renilla* luciferase activities were standardised with respect to firefly luciferase activity, and the activity in cells expressing UPF3B-Ala423 was defined as 1. Shown are average *Renilla* luciferase activities from three independent experiments. **f**, **g**: Tethering assay: Luciferase mRNA levels. HeLa cells were co-transfected with phRL-TK (**f**) or phRL-TK-10BoxB (**g**) but with phrGFP instead of pGL3-promoter. RNA was prepared 48 h later and mRNA levels were determined by qPCR. Luciferase mRNA levels were standardised with respect to GFP mRNA levels, and luciferase mRNA in cells expressing UPF3B-Ala423 was defined as 1. Shown are average luciferase mRNA levels from four independent experiments. Error bars indicate standard deviations, asterisks indicate values significantly different from luciferase activity or mRNA levels in the presence of UPF3B (one-way ANOVA followed by Dunnett's test; *P* < 0.05).

Mutation in the *UPF3B* gene located on chromosome Xq24 has been implicated in X-linked intellectual disability (XLID), autism and schizophrenia. Nonsense and missense mutations in *UPF3B* have been found in several families with syndromic and non-syndromic XLID (Table 1, Fig. 1a, Additional file 1: Figure S1) [1–6]. Several subjects in these families also display autistic features. In addition, mutation in *UPF3B* is described in schizophrenia [5]. Nonsense mutations introduce a premature termination codon, leading to a loss of UPF3B expression, most likely because they turn UPF3B mRNA into a target for nonsense-mediated mRNA decay (NMD) [2, 3]. The effect of missense mutations, which cause amino acid substitutions, upon UPF3B activity is not yet understood.

**Table 1:** *UPF3B* mutations linked to neurodevelopmental disorders

	*Mutation*	*Open Reading Frame*	*Phenotype*	*UPF3B protein*
*Family 1 [2]*	*674_677delGAAA* ^*1*^	Arg225fs*20 ^*1*^	*FG syndrome (MIM 305450), subjects with autistic features*	
*Family 2 [2]*	*867_868delAG* ^*1*^	Gly290fs*2 ^*1*^	*Lujan-Fryns syndrome (MIM 309520)*	
*Family 3 [2]*	*1288C > T* ^*1*^	Arg430* ^*1*^	*Lujan-Fryns syndrome, subjects with autistic features*	
*Family 4 [2]*	*478 T > G* ^*1*^	*Tyr160Asp* ^*1*^	*non-syndromic XLID, subjects with autistic features*	*UPF3B-Asp160*
*Family T13 [3]*	*1081C > T* ^*2*^	Arg361* ^*2*^	*non-syndromic XLID*	
*Family S98 [3]*	*1103G > A* ^*1*^	*Arg368Gln* ^*1*^	*Lujan-Fryns syndrome,* *also diagnosed as borderline ID with autism*	*UPF3B-Gln355* ^*2*^
*Family N37 [3]*	*1136G > A* ^*1*^	*Arg379His* ^*1*^	*non-syndromic XLID*	*UPF3B-His366* ^*2*^
*1 Family [1]*	*683_686delAAGA*	Gln228fs*18	*Childhood onset schizophrenia, ASD, ADHD*	
*1 Family [4]*	*c697_698delAG*	Arg233fs*30	*Developmental delay, ASD*	
*1 patient [5]*	*764G > A* ^*1*^	*Arg255Lys* ^*1*^	*schizophrenia*	*UPF3B-Lys255*
*1 Family [6]*	*1288C > T* ^*1*^	Arg430* ^*1*^	*non-syndromic XLID*	

^1^Location of mutation based on UPF3B transcript variant 1/isoform 1 (GenBank:NM_080632; GenBank:NP_542199). ^2^Location of mutation based on UPF3B transcript variant 2/isoform 2 (GenBank:NM_023010; GenBank:NP_075386). Note that transcript variant 1 starts to deviate from transcript variant 2 at nucleotide 808, and isoform 1 from isoform 2 at amino acid 269. This difference is due to skipping of an exon (808–846) in the NM_080632 open reading frame

UPF3B protein acts in the NMD pathway which has important dual roles in prevention of synthesis of truncated proteins and in regulation of gene expression. NMD targets transcripts in which translation is arrested at a premature termination codon for degradation (for review see [7, 8]). Transcripts containing premature termination codons arise for example from genes with nonsense mutations or are produced by alternative splicing [9, 10]. In addition, NMD has an important function in regulating the expression of genes with specialised regulatory features such as mRNA upstream open reading frames or long 3’ untranslated regions. Further to its role in NMD, UPF3B also promotes mRNA translation by a little understood mechanism [11].

Loss of UPF3B protein function does not fully abolish NMD activity. Vertebrates have a second *UPF3* gene termed *UPF3A* (Additional file 1: Figure S1). The contribution of *UPF3A* to NMD in the presence of *UPF3B* appears to be minor and is poorly understood [12]. However in cells lacking UPF3B protein, UPF3A protein levels are elevated and the low level of selected NMD substrates is maintained [13], indicating that UPF3A protein is at least in part able to compensate for a loss of UPF3B protein. In patients with *UPF3B* nonsense mutation the lack of *UPF3B* expression is therefore most likely due to the action of the UPF3A protein.

Normally, in situations that lead to NMD UPF1 is recruited together with the peptide release factor eRF3 to a ribosome stalled at a premature termination codon. UPF3B and UPF2 regulate UPF1 function [14]. UPF3B interacts with the exon junction complex (EJC), a protein complex deposited at exon joints, and with UPF2. UPF2 and/or UPF3B then interact with UPF1 at the stalled ribosome, and these interactions are important for the regulation of UPF1 activity [15–17]. UPF2 promotes phosphorylation of UPF1 by the kinase SMG1, leading to the recruitment of mRNA decay factors and resulting in mRNA degradation.

The missense mutations described in patients with neurodevelopmental disorders are located within protein domains of unknown function. UPF3B interacts with UPF2 via an N-terminal RNP fold located between amino acids 52–137 (Fig. 1a) and the EJC complex interaction domain in UPF3B has been mapped to amino acids 421–434 [16, 18, 19]. The missense mutations in *UPF3B* shown in Table 1 are not in these regions but are located in highly conserved parts of the protein (Fig. 1a, Additional file 1: Figure S1). Tyr160 is conserved in vertebrate and fly UPF3B proteins, human UPF3A and the *C. elegans* UPF3 homologue Smg-4. The other amino acids affected, Arg255, Arg355 and Arg366 lie in regions conserved from mammals to birds. The mutations lead to non-conservative amino acid changes of Tyr160 to Asp, Arg355 to Gln and Arg366 to His while the Arg255 to Lys substitution is a conservative amino acid change.

UPF3B protein is implicated in neuronal development. In cells depleted of UPF3B by RNAi-mediated gene knockdown or in lymphoblastoid cell lines from patients with mutations in UPF3B the expression of genes involved in neuronal development is de-regulated [13, 20]. Recent findings link NMD to neuronal differentiation and axon guidance [21, 22]. For example, knockdown of UPF3B in cultured hippocampal neurons affects the expression of NMD targets and alters neuronal outgrowth [21]. It is notable that in addition to UPF3B, other NMD genes including UPF2 and SMG6 have been implicated in neuro-developmental disorders [23].

Here we demonstrate using tethered function assays that missense mutations associated with neurodevelopmental disorders alter the function of UPF3B protein in mRNA metabolism. Using Green Fluorescent Protein (GFP)-tagged UPF3B proteins, we exclude a change in cellular localisation as a mechanism. UPF3B and UPF1 protein are down-regulated upon induction of neuronal differentiation of neural stem cells. Expression of mutant UPF3B proteins that subtly affect the mRNA levels of selected NMD substrates disturbs neuronal differentiation, specifically the branching of neurites. Neurite branching was similarly disturbed by exposure to the NMD inhibitor Amlexanox. This work indicates that despite the down-regulation of NMD factors, neuronal differentiation relies critically on fully functional NMD machinery. It contributes to our understanding of the effect of UPF3B missense mutations in patients and indicates that inhibition of the NMD pathway considered for the treatment of genetic disease caused by nonsense mutations may affect the development and function of neuronal networks.

## Results

### Missense mutations affect UPF3B function in mRNA decay

Several independent missense mutations in *UPF3B* have been reported in patients with neurodevelopmental disorders (Fig. 1a, Table 1). We wished to determine whether these mutations contribute to the disorders by affecting the function of UPF3B protein in mRNA metabolism. Tethered function assays (Fig. 1b) involving the artificial binding of a protein to mRNA are an established tool for the analysis of the function of NMD factors in living cells. Using this approach it has been shown previously that tethering UPF3B to mRNA leads to the destabilisation of the target mRNA [11, 24] and that interactions with UPF2 and exon junction complex components are important for the function of UPF3B in NMD [11, 18].

Tethering assays were performed in HeLa cells by expressing N peptide-UPF3B fusion proteins and *Renilla* luciferase mRNA with 10 BoxB RNA elements located in the 3’ untranslated region (Test RNA, expressed from phRL-TK-10BoxB). UPF3B is tethered to the reporter mRNA by the highly specific interaction between N peptide and BoxB elements in the mRNA (Fig. 1b). Control reactions to identify any effects of UPF3B that are not directly linked to its tethering to *Renilla* mRNA were performed with *Renilla* luciferase mRNA that lacks the BoxB elements and is therefore unable to bind to N peptide-UPF3B fusion proteins (Control RNA, expressed from phRL-TK).

The effects of missense mutations on UPF3B protein function was examined by comparing the activities of wild type UPF3B protein, of the 4 variants UPF3B-Asp160, UPF3B-Lys225, UPF3B-Gln355, UPF3B-His366 identified in patients with neurodevelopmental disorders (Table 1) and of UPF3B-Ala423 with the synthetic inactivating mutation Arg423Ala that was shown previously to be inactive in tethered function assays [18]. The proteins were expressed at similar levels in HeLa cells (Fig. 1c). Tethered function assays were performed by transfection of HeLa cells with the plasmid expressing either Test or Control *Renilla* luciferase mRNA and a plasmid for UPF3B protein expression. In addition, cells were transfected with pGL3-promoter expressing firefly luciferase as a reference, to allow for comparison between different transfections. The assays with Control *Renilla* mRNA in Fig. 1d show that expression of the *Renilla* luciferase mRNA lacking the BoxB elements was largely unaffected by the sequence differences between the UPF3B proteins, with *Renilla* luciferase activities varying from the control up to 35 %. In tethering assays performed with Test *Renilla* luciferase mRNA with BoxB sequences, *Renilla* luciferase activity was highest when the inactive form UPF3B-Ala423 was tethered. For presentation in the graphs this was defined as 1 (Fig. 1e). Tethering of wild type UPF3B caused an approximately ten-fold reduction of *Renilla* luciferase activity, reflecting the earlier observation that tethering of UPF3B protein, but not UPF3B-Ala423 protein causes the mRNA to decay [18]. Tethering of UPF3B proteins found in patients with neurodevelopmental disorders resulted in three- to five-fold higher *Renilla* luciferase activity than when wild type UPF3B protein was tethered. This indicates that the function of these proteins in NMD is impaired.

To examine directly the effect of the mutations on the ability of UPF3B protein to control mRNA levels we compared *Renilla* luciferase mRNA levels using a reverse transcription/qPCR approach. Experiments were performed in HeLa cells as described above, except that phrGFP-C expressing GFP was used as a reference instead of pGL3 promoter. The expression of the various UPF3B proteins did not affect the levels of the Control *Renilla* luciferase mRNA significantly (Fig. 1f). Tethering of wild type UPF3B protein to Test *Renilla* luciferase mRNA led to an approximately 20-fold reduction of mRNA compared to when UPF3B-Ala423 protein was tethered (Fig. 1g). Significantly, tethering of the other UPF3B proteins led to six- to ten-fold higher luciferase mRNA levels than when wild type UPF3B was tethered. Together, these experiments demonstrate that mutations in UPF3B linked to neurodevelopmental disorders impair the activity of UPF3B protein in NMD.

### Downregulation of NMD factors UPF3B and UPF1 during neuronal differentiation

**Fig. 2:**
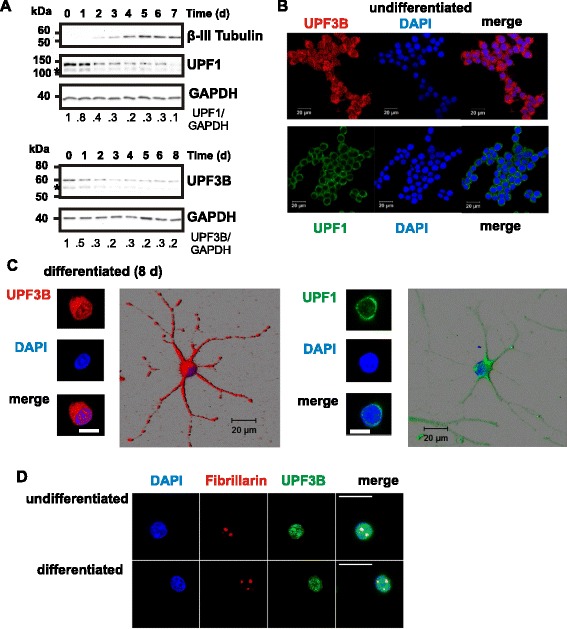
NMD factors UPF1 and UPF3 localise to neurites while protein levels are downregulated during neuronal differentiation. **a**. Neural stem cells were differentiated by the addition of forskolin and retinoic acid. Protein samples were prepared before (day 0 (d 0)) and at the indicated time (d, days) after induction of differentiation. Equal amounts of protein were subjected to 8 % SDS PAGE and analysed for β-III Tubulin, UPF3B and UPF1 protein levels by Western blotting. Samples were probed for GAPDH protein as loading control. Proteins with faster mobility indicated by asterisk reflect the behaviour of UPF1 and UPF3B and are likely to be alternate isoforms. The relative changes of UPF3B and UPF1 protein levels were quantitated using GAPDH as reference, with levels at day 0 defined as 1. The mobility of relevant molecular mass markers is indicated. **b**. UPF1 and UPF3B protein localisation in undifferentiated HCN-A94 cells. Cells were stained with DAPI (blue) and with either anti-UPF3B antibody (red) or anti-UPF1 antibody (green) were analysed by confocal microscopy. Shown are separate channels and merged images from a single section. **c**. UPF3B and UPF1 localisation in neurons. Neural stem cells differentiated for 8 days and then stained with DAPI and either anti-UPF3B antibody or anti-UPF1 antibody were analysed by confocal microscopy. Shown are separate channels and merged images from a single section through the nucleus and 3 days renderings of corresponding Z-stacks. The scale bars represent 20 μm. Note that UPF3B and UPF1 are detected in neurites and UPF1, but not UPF3B, largely is excluded from the nucleus. **d**. Nucleolar localisation of UPF3B. Undifferentiated or for 8 day differentiated neural stem cells were stained with anti-UPF3B antibodies, anti-Fibrillarin antibodies and DAPI and then analysed by confocal microscopy. Sections shown focus on the nuclear compartment. The scale bars represent 20 μm. Note that UPF3B is enriched in nucleoli stained by anti-Fibrillarin antibodies

We wished to examine the effect of the expression of mutant UPF3B proteins on neuronal differentiation. We decided to use HCN-A94 rat neural stem cells as a model system. These cells have maintained the ability to form neurons when grafted into adult brain and can be induced to differentiate into neurons *in vitro* [25, 26]. Figure 2a shows that the induction of neuronal differentiation leads to the expression of the neuronal marker β-III Tubulin. Expression of β-III Tubulin in uninduced cells (day 0) was below the level of detection by Western blotting, but was detected from day 2 onward, confirming the neuronal differentiation of these cells. Neuronal differentiation was reflected also in the formation of neurites that increased in complexity over time (see Fig. 5). We examined the expression of NMD factors UPF1 and UPF3B during the differentiation process (Fig. 2a). The levels of both proteins decreased rapidly after induction of differentiation and were at between 10 % and 20 % of the levels in undifferentiated cells after seven to eight days of differentiation. We observed also a reduction of UPF3B mRNA levels during differentiation (Additional file 1: Figure S2). In conclusion, UPF1 and UPF3B are down-regulated during neuronal differentiation.

**Fig. 3:**
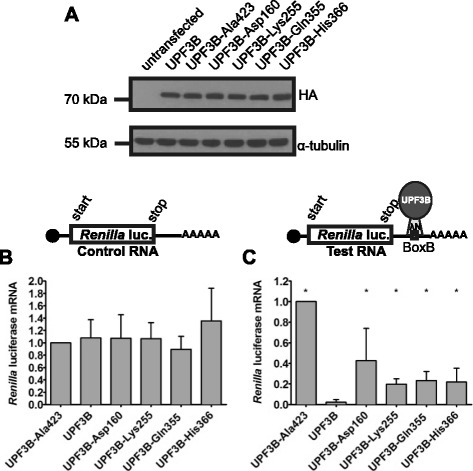
Mutations in *UPF3B* impair UPF3B protein NMD activity in neural stem cells. **a**. Expression of λN-HA-UPF3B proteins. Neural stem cells were transfected with pCI-λN-HA-UPF3B expression constructs and protein expression was analysed 48 h after transfection by 10 % SDS PAGE followed by detection by Western blotting. UPF3B proteins and α-tubulin were detected using anti-HA and anti-tubulin antibodies. **b**, **c**. Tethering assays. **b**. Neural stem cells were transfected with the control plasmid phRL-TK, the reference plasmid phrGFP-C and the various pCI-λN-HA-UPF3B expression constructs. 24 h after transfection, differentiation was induced. RNA was prepared 48 h after transfection and analysed by qPCR as described. **c**. Neural stem cells were transfected, treated and mRNA levels analysed as in B except that phRL-TK was replaced with phRL-TK-10BoxB. For the graphs, *Renilla* luciferase mRNA levels were standardised with respect to GFP mRNA levels, and *Renilla* luciferase mRNA levels in cells expressing UPF3B-Ala423 was defined as 1. The graphs show average *Renilla* luciferase mRNA levels from three independent experiments, error bars indicate the standard deviation. Asterisks indicate values significantly different from luciferase mRNA levels in the presence of UPF3B (one-way ANOVA followed by Dunnett's test; *P* < 0.05)

### Localisation of UPF3B and UPF1 in neural stem cells

We examined the cellular localisation of these proteins by staining neural stem cells with anti-UPF1 and anti-UPF3B antibodies. In replicating cells, UPF1 was found in the cytoplasm. UPF3B on the other hand was detected in the cytoplasm and nucleus where it was enriched in small dots (Fig. 2b). A more detailed analysis of UPF3B nuclear localisation revealed that the nuclear signal was generally diffuse but concentrated in the nucleoli detected by staining with anti-Fibrillarin antibody (Fig. 2d). After eight days of neuronal differentiation, cellular distributions of UPF1 and UPF3B were similar to undifferentiated cells: UPF1 was detected in the somatic cytoplasm and throughout the neurites while UPF3B was detected in the somatic cytoplasm, in neurites and in the nucleus, where it was enriched in nucleoli (Fig. 2c, d).

### Differentiating neural stem cells are competent for UPF3B-promoted NMD

We wished to determine whether differentiating neural stem cells maintain the ability to mediate UPF3B-promoted NMD, and whether this would be sensitive to mutations in UPF3B. First we determined that the proteins were expressed similarly in neural stem cells (Fig. 3a). Tethering assays were then performed with wild type UPF3B, UPF3B-Ala423 and the UPF3B mutants from patients by transfection of neural stem cells with the appropriate plasmid combinations followed by induction of differentiation for two days (Fig. 3b). The analysis by reverse transcription followed by qPCR showed that Test *Renilla* luciferase mRNA levels were reduced approximately 40-fold when wild type UPF3B was tethered compared to when UPF3B-Ala423 was tethered. This was specific for tethering of UPF3B as in reactions with Control *Renilla* luciferase RNA, mean mRNA levels varied by only 7 %. This indicates that differentiating neural stem cells maintain the ability to perform UPF3B-promoted NMD. Tethering of the mutant UPF3B proteins led to *Renilla* luciferase mRNA levels between 10- and 20-fold higher than with wild type UPF3B protein. These significant effects on *Renilla* luciferase mRNA levels were caused by binding of UPF3B proteins to the Test mRNA as in the samples with Control *Renilla* luciferase mRNA the differences in mRNA levels were small and means from three independent experiments varied by only 35 %. Together these experiments indicate that differentiating neural stem cells are able to undergo UPF3B-promoted NMD, and that NMD in these cells is sensitive to UPF3B protein variants found in subjects with neurodevelopmental disorders.

**Fig. 4:**
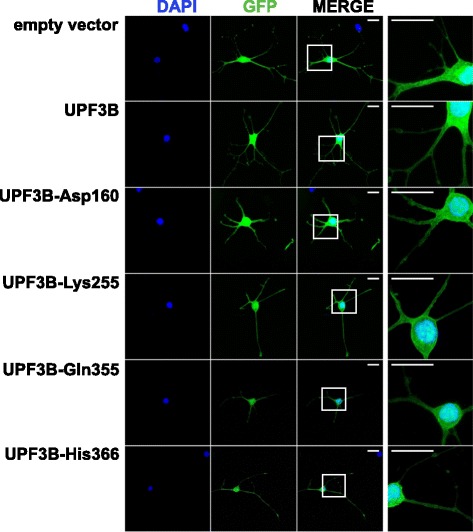
Mutations in *UPF3B* do not affect its cellular distribution in neurons. Neural stem cells were transfected with the pEGFP-C3 expressing GFP only (empty vector) or a pEGFP-C3 derivative expressing one of the UPF3B forms with an N-terminal GFP tag. Cells were diluted and re-plated after 24 h, and then differentiated for 6 days prior to analysis by confocal microscopy. Shown are separate Z-stack projections of GFP and DAPI channels and of the merged channels. The origin region of the enlarged section is indicated. The scale bar represents 20 μm

### Missense mutations in UPF3B do not affect cellular localisation in neurons

To determine whether the reduced activity of the UPF3B proteins found in subjects with neurodevelopmental disorders is linked to a restriction in cellular localisation we expressed the various UPF3B proteins with an N-terminal GFP tag in neural stem cells and analysed the localisation of GFP-tagged proteins by confocal microscopy. In neurons after six days of differentiation all UPF3B proteins were detected in the somatic cytoplasm, in neurites and in the nucleus (Fig. 4). Similar to the localisation of endogenous UPF3B (Fig. 2), nuclear GFP-tagged UPF3B was enriched in nucleoli and missense mutations in UPF3B did not significantly alter the pattern of nuclear and nucleolar localisation (Additional file 1: Figure S3). Thus the reduction of activity in NMD caused by the mutations is not linked to an obvious effect on cellular localisation. During these experiments we noticed that the pattern of neurites formed during differentiation appeared less complex in cells expressing the mutant UPF3B GFP fusion proteins, indicating that the expression of these proteins may have an effect on neuronal differentiation.

**Fig. 5:**
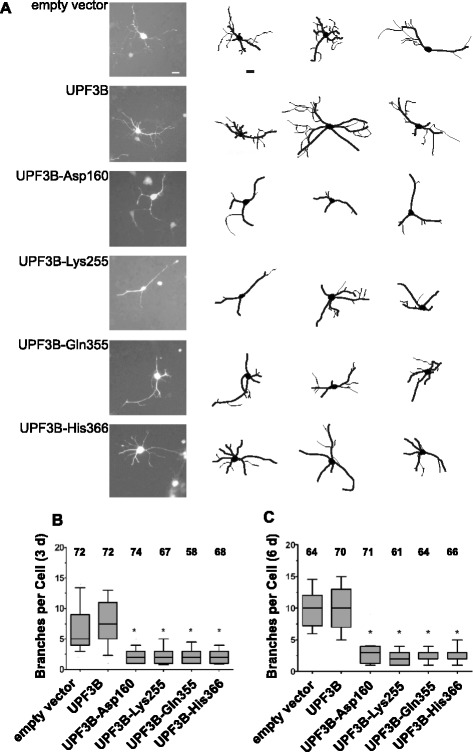
Expression of mutant UPF3B proteins affects neuronal differentiation of neural stem cells. Neural stem cells were transfected with pIRES-GFP (empty vector) or pIRES-GFP derivatives expressing the indicated UPF3B proteins. Differentiation was induced after 24 h, and analysed after 3 days and 6 days of differentiation by microscopy. Transfected cells were identified and visualised by GFP fluorescence. The analysis of neuron differentiation was performed as described in Methods. **a**. Representative pictures of neurons with tracing, and tracing of 2 additional neurons after 6 days of differentiation illustrating a reduction in branching in neurons expressing mutant UPF3B proteins. The scale bars represent 20 μm. Tracings are to scale with primary neurites traced with thicker lines then branches protruding from primary neurites. **b**, **c**. Branching was analysed after 3 days and 6 days as described and results are presented as box plots. The box plots show the median, with boxes spanning 25^th^- 75^th^ percentiles and the crossbars indicating the 10^th^ and 90^th^ percentiles. Experiments were done independently 3 times with similar outcomes and the data were combined for presentation. Numbers of cells analysed are indicated. Asterisks indicate significantly reduced branching in neurons expressing mutant UPF3B proteins compared to neurons transfected with the empty vector (Kruskal Wallis test followed by Dunn's multiple comparison test; *P* < 0.05)

### Expression of UPF3B with missense mutations disturbs neuronal differentiation of neural stem cells

We therefore wished to establish whether the expression of the UPF3B protein variants found in patients with neurodevelopmental disorders affects neuronal differentiation. To avoid artefacts caused by the expression of the UPF3B proteins fused to GFP we inserted the UPF3B protein open reading frames into pIRES-GFP, resulting in constructs expressing UPF3B proteins and GFP separately from a bicistronic transcript with an internal ribosome entry site (IRES) located between the UPF3B and GFP open reading frames. After transfection, cells were induced to differentiate. Transfected cells were identified and analysed by fluorescence microscopy after a further three or six days. Examples of the appearance of neurons transfected with the various constructs after six days of differentiation are shown in Fig. 5a. While neurons transfected with the empty vector pIRES-GFP and with pIRES-GFP expressing UBP3B displayed a complex branching pattern, this appeared much reduced in the neurons expressing the mutant proteins. To quantify effects on differentiation, neurite outgrowth was traced and analysed using NeuronJ software. For this analysis, outgrowths directly from the soma were categorised as primary neurites, while secondary and tertiary neurites arising from primary neurites were categorised as branches. These data are shown as box-and-whisker plots in Fig. 5 and Additional file 1: Figure S4. There was no significant difference in the numbers of primary neurites, primary neurite length, branching and the length of the branches between cells transfected with the empty vector and cells expressing wild type UPF3B. This indicates that expression of UPF3B protein did not have a significant effect on neuronal differentiation. While the number of primary neurites per cell was largely unaffected by the presence of the mutant proteins we detected subtle differences in the length of primary neurites and of branches that were however not statistically significant (Additional file 1: Figure S4). In contrast, the number and complexity of branches was reduced significantly in cells expressing UPF3B proteins encoded by genes with mutations occurring in neurodevelopmental disorders (Fig. 5). After three days of differentiation, neurite branching in cells expressing these mutant UPF3B proteins was approximately two- to three-fold reduced compared to cells transfected with the empty vector, or the vector expressing wild type UPF3B protein (Fig. 5). This effect increased with time, leading to approximately a three- to six-fold reduction in branching after six days of differentiation. In summary, the expression of UPF3B proteins found in patients with neurodevelopmental disorders, but not wild type UPF3B protein, disturbs neuronal differentiation, specifically the branching of neurites. This indicates that the expression of these UPF3B proteins affects neuronal differentiation and demonstrates that full UPF3B function is critical for this process.

### Functional NMD is important for neuronal differentiation of neural stem cells

**Fig. 6:**
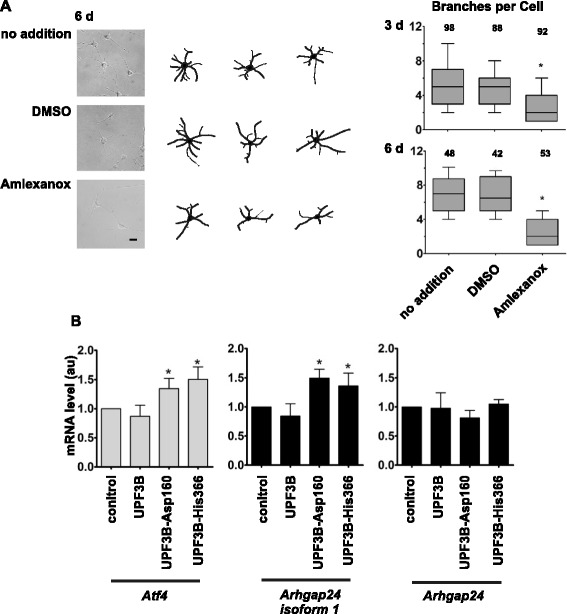
A functional NMD pathway is important for neuronal differentiation. **a**. Treatment with NMD inhibitor Amlexanox affects neuronal differentiation of neural stem cells. Neural stem cells were differentiated in the presence of DMSO or 10 μM Amlexanox or without any further addition. Neurons were analysed 3 days and 6 days after induction of differentiation as described. Shown are representative pictures of neurons with tracing, and tracing of 2 additional neurons after 6 days of differentiation illustrating a reduction in branching in Amlexanox treated neurons. The scale bar represents 20 μm. Results are plotted showing the median, with boxes spanning 25^th^- 75^th^ percentiles and the crossbars indicating the 10^th^ and 90^th^ percentiles. Experiments were done independently 3 times with similar outcomes and the data were combined for presentation. Numbers of cells analysed are indicated. The Asterisk indicates that branching of Amlexanox treated neurons is significantly reduced compared to neurons differentiated without addition any addition or DMSO only (Kruskal Wallis test followed by Dunn's multiple comparison test; *P* < 0.05). **b**. Expression of mutant UPF3B proteins that affect neuronal differentiation causes an increase in mRNA levels of selected NMD substrates. Neural stem cells were transduced with lentivirus without insert (control) or expressing either UPF3B, UPF3B-Asp160 or UPF3B-His366 protein. 24 h after transduction, differentiation was induced. RNA was isolated after six days of differentiation and gene expression was analysed by reverse transcription followed by quantitative PCR. mRNA levels of NMD substrates *Atf4* and *Arhgap24* isoform 1, and of transcripts encoding all shorter *Arhgap24* isoforms not subject to NMD control were measured and standardised using *Gapdh* mRNA as reference. *Atf4* and *Arhgap24* mRNA levels in cells transduced with the control virus were set as 1. Asterisks indicate values significantly increased compared to levels in cells transduced with the control virus (one-way ANOVA followed by Dunnett's test; *P* < 0.05)

To determine whether the effect on neuronal differentiation observed in Fig. 5 is compatible with a defect in NMD we treated cells with the NMD inhibitor Amlexanox. Lejeune and colleagues have shown that this compound is able to inhibit UPF3B-induced mRNA decay but does not act by inhibiting translation, a non-specific mechanism to stabilise mRNAs [27]. Neural stem cells were differentiated in the presence of Amlexanox, and for comparison in the presence of the solvent DMSO or without any further addition. Analysis of cells after three and six days of differentiation revealed that the number of branches per cell was reduced two-fold in the presence of Amlexanox (Fig. 6a), similar to the effect of the expression of UPF3B proteins encoded by genes with mutations occurring in neurodevelopmental disorders (Fig. 5). In addition we observed that treatment with Amlexanox caused a subtle but significant reduction in the number of primary neurites per cell and, after extended incubation, a reduction in branch length (Additional file 1: Figure S5).

Next we wished to examine whether expression of mutant UPF3B proteins affects NMD in neurons. We transduced neural stem cells with lentivirus constructs expressing wild type UPF3B protein and the mutant forms UPF3B-Asp160 and UPF3B His-366 or the lentivirus backbone without any additional gene and subsequently induced differentiation. We decided to examine the effect of UPF3B-Asp160 and UPF3B -His366 expression as representatives of proteins with mutations located in the N-terminal and the C-terminal halves of UPF3B, respectively. After six days of differentiation, total RNA was isolated and the levels of *Atf4*, *Arhgap24* and for comparison of *Gapdh* mRNA were determined. Both *Atf4* and *Arhgap24* transcripts contain upstream open reading frames (uORFs) which are classic features of mRNAs regulated by the NMD process. Human and mouse ARHGAP24 isoform 1 is encoded by mRNAs with upstream open reading frames (uORFs) that are subject to nonsense-mediated decay [20]. Rat mRNA sequences GenBank:FQ225302, GenBank:CB582621 and GenBank:CB609913 encode rat ARHGAP24 isoform 1. By alignment of these mRNAs and human and mouse ARHGAP24 isoform 1 mRNAs with rat genomic DNA (RGSC Genome Assembly 5.0) we identified a similar uORF in the rat genome upstream of the annotated *Arhgap24* gene (chromosome 14 positions 8736874–8737122). ARHGAP24 isoform 1 mRNA is upregulated in subjects with *UPF3B* mutations and overexpression of ARHGAP24 isoform 1 protein has been shown to affect neuronal branching [20]. *Atf4* is a well-established target of NMD involved in neuronal plasticity [28, 29]. Figure 6b shows that the levels of both *Atf4* and *Arhgap24* isoform 1 mRNAs were elevated in cells expressing UPF3B-Asp160 protein or UPF3B His-366 protein compared to cells expressing UPF3B or control-transduced cells. *Arhgap24* also expresses shorter isoforms lacking the N-terminal PH domain of ARHGAP24 isoform 1 and the uORF in the 5’ UTR. To examine whether the effect on *Arhgap24* expression was limited to NMD substrates we measured expression using a qPCR assay able to detect all known and predicted *Arhgap24* transcripts. Using this assay we did not observe a significant difference in *Arhgap24* transcript levels between cells expressing UPF3B-Asp160 protein or UPF3B His-366 protein and cells expressing UPF3B or control-transduced cells. This indicates that the expression of UPF3B-Asp160 protein or UPF3B His-366 proteins specifically affects NMD targets. Together, these observations indicate that the effects of the missense mutations in UPF3B on neuronal development are caused by an impairment of the NMD process.

## Discussion

We have shown that UPF3B proteins with missense mutations found in patients with autism, schizophrenia and XLID are functionally impaired in NMD. We have used tethering assays widely used to study the function of NMD proteins to assess the ability of mutant UPF3B proteins from patients with these disorders to participate in this process (Figs. 1 and 3). The activity of these UPF3B proteins in NMD was reduced significantly, but not inhibited fully. We performed this analysis in HeLa cells and in HCN-A94 cells, a model for neuronal differentiation. The results in these two cell types were similar although there were differences in the extent of the effects of specific mutations on the NMD process. It is however difficult to directly compare these experiments as levels in NMD factors in neurons are reduced upon induction of differentiation (Fig. 2).

UPF1 in replicating neural stem cells was localised mainly in the cytoplasm, as reported previously for other cell types [24, 30] and remained so in differentiated neurons (Fig. 2). UPF3B in undifferentiated and differentiated neural stem cells was detected in both the nucleus where it was enriched in nucleoli, and in the cytoplasm (Fig. 2). Nucleolar localisation of UPF3 was observed in plants, and human UPF3B was identified as a nucleolar component by proteomic analysis [31, 32]. In HeLa cells, UPF3B is localised to the nucleus and cytoplasm with strong nuclear localisation, reflecting that UPF3B is a nucleo-cytoplasmic shuttling protein that performs its function in NMD in the cytoplasm [24, 33, 34]. The UPF3B localisation patterns observed in the brain is more complex and changes during development [21]. For example, UPF3B is being largely excluded from the nucleus in neural progenitor cells during early stages of mouse embryonic development while at later stages the protein clearly localises to the nucleus in the same cell type.

We observed that the levels of NMD factors UPF1 and UPF3B are down-regulated during neuronal differentiation of neural stem cells (Fig. 2), similar to observations by others in mouse neural stem cells [21, 35]. Down-regulation of UPF1 and concomitant reduction of NMD activity was linked to changes in gene expression favouring neuronal differentiation of mouse neural stem cells, providing a paradigm for the role of the NMD process during neuronal differentiation [35].

Using GFP-tagged UPF3B proteins we showed that cellular localisation was not affected by the mutations (Fig. 4 and Additional file 1: Figure S3), in agreement with a previous analysis of the localisation of UPF3B-His366 and UPF3B-Gln355 in primary neurons [3]. This indicated that the effect of the missense mutations on NMD was not due to a change in cellular localisation of UPF3B proteins.

Our findings indicate that UPF3B protein, through its function in NMD, is important for neuronal differentiation. The neuronal differentiation of neural stem cells was disturbed by the expression of UPF3B proteins from patients with neurodevelopmental disorders. Specifically the branching of neurites was reduced significantly (Fig. 5). This can be linked to a defect in NMD; treatment of cells with the NMD inhibitor Amlexanox similarly affected neurite branching, and the expression of two mutant UPF3B proteins, UPF3B-Asp160 and UPF3B-His366, led to a subtle but significant increase of the expression of the NMD substrates *Atf4* and *Arhgap24* (Fig. 6). *Arhgap24* is of particular interest as aberrant expression of *Arhgap24* in primary hippocampal neurons has been shown to cause a reduction of neuronal arborisation [20]. The observation that X chromosome inactivation in female carriers in families with UPF3B missense mutations is skewed towards inactivation of the chromosome carrying the *UPF3B* locus with the mutations is compatible with that the expression of these proteins may be detrimental [2, 3].

It will be interesting to explore how the mutations in *UPF3B* would affect UPF3B activity. The four mutations we analysed are not in one of the regions known to be important for UPF3B function; the RNP motif (Fig. 1, amino acids 52–137) and the exon junction complex interaction domain (amino acids 421–434), instead they are located between these two elements (Fig. 1, Additional file 1: Figure S1). Structural information is available for the UPF3B N-terminus and the C-terminus with the exon junction complex interaction domain, but a complete structure encompassing the regions containing the mutations is to our knowledge not currently available [16, 19]. We are therefore unable to make predictions about the effects of the mutations on UPF3B protein structure. However it is possible that these mutations, by changing the secondary or tertiary structure of UPF3B protein, affect the function of the domains involved in protein-protein interaction with UPF2 protein and the EJC, respectively. Interestingly in patients expressing UPF3B-Asp160, UPF3A is up-regulated similar to the situation in cells from patients that lack UPF3B expression [13, 20]. As UPF3A protein levels are controlled and kept low by competition with UPF3B for binding to UPF2 this suggests that the replacement of Tyr160 by Asp in UPF3B protein may attenuate the UPF3B-UPF2 interaction.

Post-mortem studies and mouse models have linked neurodevelopmental disorders to the complexity of neuronal branching. Decreased dendritic complexity of cortical neurons has been demonstrated post-mortem in schizophrenia [36, 37]. Post-mortem examination of neurons from patients with Rett syndrome revealed structural changes including a reduction in dendrites of cortical neurons [38]. Reduced complexity of dendrites has been observed also for hippocampal neurons of a mouse model for Rett syndrome and for cortical neurons in a mouse model for Timothy syndrome [39, 40]. Our findings implicate UPF3B and the NMD pathway in the control of dendrite complexity.

In conclusion, our observations indicate that despite the down-regulation of NMD factors during neuronal differentiation in neural stem cells, fully functional NMD machinery is critical for appropriate differentiation. Missense mutations in UPF3B found in patients with neurodevelopmental disorders may contribute to the development of these disorders by an impairment of UPF3B activity in the NMD process that results in aberrant gene expression affecting neuronal differentiation. Normalisation of NMD might be a route to ameliorate the phenotype. Inhibition of NMD is being considered as strategy to treat genetic diseases caused by nonsense mutations, possibly in combination with compounds such as Ataluren that facilitate the read through of nonsense codons by the ribosome [41]. This approach may impact on the development and function of neuronal networks as inhibition of nonsense-mediated decay is likely to interfere with the expression control of genes with critical functions in neurons and other cell types.

## Methods

### Cell culture

HeLa cells were maintained in DMEM media supplemented with 10 % fetal bovine serum. HCN-A94 adult rat hippocampal neural stem cells were obtained from Fred Gage (Salk Institute, La Jolla, CA, USA) and were maintained as described [25] in DMEM/F12 medium with N2 supplement and 20 ng/ml FGF-2 (Peprotech). Neuronal differentiation was induced in DMEM/F12 medium with N2 supplement (Life Technologies) and with 1 μM retinoic acid and 5 μM forskolin [26]. All cells were cultivated at 37 °C in a 5 % CO_2_ atmosphere. Amlexanox (Tocris Bioscience) was dissolved in DMSO and applied to cells together with retinoic acid and forskolin.

### Nucleic acids

pGL3-promoter expressing firefly luciferase was from Promega and phrGFP-C was from Stratagene/Agilent. Plasmids pCI-λNhUPF3B and pCI- λNhUPF3BArg423Ala expressing the human UPF3B isoform 2 (GenBank:NP_075386) and the UPF3BArg423Ala protein were a generous gift of Niels Gehring (University of Cologne, Germany) [18]. An HA tag was introduced into these plasmids in the XhoI site between the λN and UPF3B domains, producing pCI-λN-HA-UPF3B and pCI-λN-HA-UPF3B-Ala423. Other mutations were introduced into pCI-λN-HA-hUPF3B by site-directed mutagenesis using the QuikChange Lightning Site-Directed Mutagenesis Kit (Agilent Technologies). This resulted in plasmids pCI-λN-HA-UPF3B-Asp160, pCI-λN-HA-UPF3B-Lys225, pCI-λN-HA-UPF3B-Gln355, pCI-λN-HA-UPF3B-His366 and pCI-λN-HA-UPF3B-Ala423, respectively. Sequences are available on request. UPF3B fragments were excised from the pCI-λN-HA plasmids using XhoI and SalI and inserted into pEGFP-C3 (Clontech Laboratories) plasmid cleaved with the same enzymes. This resulted in plasmids pEGFP-UPF3B, pEGFP-UPF3B-Asp160, pEGFP-UPF3B-Lys225, pEGFP-UPF3B-Gln355, UPF3B-His366 and pEGFP-UPF3B-Ala423. The UPF3B open reading frame was amplified from pCI-λN-HA-N-UPF3B plasmid using primers with BamHI and XhoI restriction sites. The amplicon was inserted into pGEM-Teasy (Promega) and then released using BamHI and XhoI and inserted into pIRES-AcGFP1 (Clontech Laboratories; abbreviated pIRES-GFP) cleaved with the same enzymes. Mutations were introduced into UPF3B by site-directed mutagenesis as described, producing plasmids pIRES-UPF3B-GFP, pIRES-UPF3B-Asp160-GFP, pIRES-UPF3B-Lys225-GFP, pIRES-UPF3B-His366-GFP and pIRES-UPF3B-Ala423-GFP. UPF3B fragments were amplified from pEGFP constructs and inserted into the EcoRI site of pCDH-EF1-MCS-IRES-GFP (Systems Biosciences, Mountain View, CA, USA) by In-Fusion cloning (Clontech Laboratories). phRL-TK-10BoxB was produced by releasing the 5BoxB element located in the 3’ untranslated region of in pRL-TK-5BoxB [42] using an XbaI digest and replacing it with an element containing 10BoxB repeats. All plasmids constructions were confirmed by Sanger dideoxy sequencing.

### Tethered function assays

For luciferase activity assays, HeLa or HCN-A94 cells were grown in 24 well plates and transfected with 0.1 μg phRL-TK-10BoxB plasmid, 0.1 μg of pGL3 promoter plasmid and with 0.7 μg of one of the six pCI-λN-HA-tagged UPF3B expression constructs. For mRNA analysis by qPCR, HeLa or HCN-A94 cells grown in 35 mm plates were transfected with 0.5 μg phRL-TK-10BoxB plasmid, 0.5 μg of phrGFP plasmid and 5 μg of one of the six pCI-λN-HA-UPF3B expression constructs. Transfections were done using either GeneTran™ (Biomiga), or Lipofectamine 2000™ (Life Technologies). Each transfection was done in triplicate, and each experiment was done at least three times. Transfections into HeLa cells were analysed after 48 h. HCN-A94 cells were induced to differentiate 12–24 h after transfection and were analysed after a further 48 h. Luciferase activity was analysed using the dual luciferase reporter assay system and a GloMaxR Luminometer (Promega). Total RNA was prepared using the Biomiga Tissue RNA Mini Kit. Samples were treated with DNase I according to the manufacturer’s instruction. cDNA synthesis was done using Superscript II reverse transcriptase (Life Technologies) and random hexamer primers (Bioline, London, UK). Quantitative PCR (qPCR) reactions were performed in a Roche LightCycler 480 qPCR system with Universal ProbeLibrary assays (Additional file 1: Table S1).

### Analysis of neuronal differentiation

HCN-A94 cells were grown in 6 well plates and transfected with pEGFP-UPF3B constructs or pIRES-UPF3B-GFP constructs. After 24 h, cells were diluted and transferred into 24 well plates. After a further 24 h, differentiation was induced. Live cell images were taken using an Axiovert 40CFL microscope with Axio Vison software (Zeiss). Pictures were converted into grayscale Tiff files and analysed using ImageJ software with NeuronJ plugin to trace cells [43]. Neurites protruding directly from the soma were classified as primary neurites. Secondary and tertiary neurites originating from neuritic processes and ending at a terminus were classified as branches.

### Protein localisation

For the localisation of GFP-UPF3B fusion proteins HCN-A94 cells were seeded in 6-well plates and the following day were transfected with 5 μg of pEGFP-C3 or derivatives expressing GFP-UPF3B fusion proteins using Lipofectamine 2000 as per manufacturer’s instructions. The following day cells were diluted in 35 mm μ-Dishes (Ibidi) and grown for 48 h or differentiated for six days. The medium was removed and cells were fixed in PBS/4 % paraformaldehyde for 10 min at room temperature and washed three times with PBS for 5 min. Cell nuclei were stained with DAPI for 5 min and then washed three times with PBS.

For detection of endogenous UPF3B and UPF1 proteins cells were fixed in ice-cold 100 % methanol at −20 °C for 15 min and washed three times in PBS for 5 min. Non-specific interactions were incubated with 5 % normal goat serum (Sigma-Aldrich) in PBS with 0.3 % Triton X-100 for staining with anti-UPF3B antibody staining and with 10 % BSA in PBS with 0.3 % Triton X-100 for staining with anti-UPF1 antibody for 1–2 h. Cells were stained with rabbit anti-UPF3B (Abgent) or goat anti-RENT1 (UPF1) (Bethyl Laboratories) antibodies in 1x PBS, 1 % BSA, 0.3 % Triton X-100 overnight at 4 °C. Then cells were washed three times in PBS and probed with goat anti-rabbit AlexaFluor 555 or donkey anti-goat AlexaFluor 488 (Life Technologies) diluted in antibody solution for 1 h and then washed three times with PBS. Cells were counterstained with DAPI for 5 min and washed three times with PBS.

For detection of Fibrillarin and UPF3B, cells were fixed in 4 % paraformaldehyde in PBS, washed three times with PBS and permeabilized in 0.1 % Triton X-100. Following 1 h blocking in 5 % normal goat serum (Sigma-Aldrich) in PBS with 0.3 % Triton X-100 cells were incubated with mouse anti-Fibrillarin antibody (1:500; Abcam) overnight at 4 °C in 1x PBS, 1 % BSA, 0.3 % Triton X-100. The next day cells were washed three times with PBS and incubated with goat anti-mouse Alexa Fluor 594 (Invitrogen) in 1x PBS, 1 % BSA, 0.3 % Triton X-100. After 1 h, cells were then washed three times with PBS, blocked for 1 h and incubated with anti-UPF3B (1:400, Abgent) in in 1x PBS, 1 % BSA, 0.3 % Triton X-100 overnight at 4 °C. Then cells were washed three times with PBS and incubated with goat anti-rabbit Alexa Fluor 888 (1:400, Invitrogen). After 1 h cells were washed three times with PBS, counterstained with DAPI and washed again three times with PBS. Images were captured using an inverted confocal microscope (Zeiss LSM 710) with 63x oil objective and analysed using ZEN (Zeiss) and ImageJ software [43]. Microscopy was performed in the Microscopy and Histology Core Facility at the University of Aberdeen.

### Western blotting

Proteins were separated using 8 % or 10 % SDS polyacrylamide gel electrophoresis. Proteins were transferred onto Hybond P membrane (GE Healthcare) and detected using either a mouse monoclonal anti-HA antibody (HA.11, Covance), a mouse monoclonal anti-α-tubulin antibody (Sigma-Aldrich), a polyclonal goat UPF1 antibody (Bethyl Laboratories), an affinity-purified rabbit polyclonal UPF3B antibody (Abgent), a mouse monoclonal GAPDH antibody (Pierce) or a rabbit monoclonal β-III Tubulin antibody (Cell Signaling Technology). Horseradish peroxidase coupled secondary anti-mouse and anti-rabbit antibodies were from Cell Signaling Technology and horse radish peroxidase coupled secondary anti-goat antibodies from Santa Cruz Biotechnology. Proteins were detected using ECL or ECL plus chemiluminescence reagents (GE Healthcare) and visualised using X-ray film and developed using an X-Omat processor (Kodak) or using a Thermo Scientific My ECL imager. The images produced were analysed using ImageJ software or Image Studio Lite software (LI-COR Biosciences).

### Analysis of NMD by qPCR

Lentivirus particles were produced by transfection of HEK-293 T cells with pCDH-EF1-MCS-IRES-GFP and UPF3B expressing derivatives together with helper plasmid mix pPACKH1 (System Biosciences). After 2 days, virus-containing supernatants were collected and applied to neural stem cells and after a further 24 h, differentiation was induced. Total RNA was prepared using a Purelink RNA kit with DNAse treatment (Life Technologies). cDNA synthesis was done using Superscript II reverse transcriptase (Life Technologies) and oligo-dT primers (Promega). Quantitative PCR (qPCR) reactions to detect *Gapdh*, *Arhgap24* and *Atf4* mRNAs were performed using a LightCycler 480 qPCR system with Universal ProbeLibrary assays (Roche) (Additional file 1: Table S1). ARHGAP24 isoform 1 (Swiss-Prot:Q5U2Z7) encoding transcripts were identified by blast search. The qPCR assay specifically detects the region encoding the ARHGAP24 isoform 1 N-terminal PH domain represented by *Arhgap24* mRNAs GenBank:CB582621 and GenBank:CB609913. A second *Arhgap*24 assay detects transcripts encoding ARHGAP24 isoform 1, the shorter isoform encoding GenBank:NP_001012032 and predicted transcripts encoding related isoforms lacking the PH domain.

## Additional file

Additional file 1:
**Supplemental Figures S1-S5, supplemental Table S1 and supplemental Methods.**

## Abbreviations

ADHD: Attention deficit hyperactivity disorder
ASD: Autism spectrum disorder
*Atf4*: Activating transcription factor 4
*Arhgap24*: Rho GTPase activating protein 24
EJC: exon junction complex
*Gapdh*: Glyceraldehyde 3-phosphate dehydrogenase
GFP: Green Fluorescent protein
IRES: internal ribosome entry site
NMD: nonsense-mediated mRNA decay
RNP: ribonucleoprotein
SMG6: SMG6 nonsense mediated mRNA decay factor
uORF: upstream open reading frames
UPF1: UPF1 regulator of nonsense transcripts homolog
UPF2: UPF2 regulator of nonsense transcripts homolog
UPF3A: UPF3 regulator of nonsense transcripts homolog A
UPF3B: UPF3 regulator of nonsense transcripts homolog B
XLID: X-linked intellectual disability
