# Supplementary material for: Full UPF3B function is critical for neuronal differentiation of neural stem cells
Source: Mol Brain. 2015 May 27;8:33. doi: 10.1186/s13041-015-0122-1 (PMC4445987; doi:10.1186/s13041-015-0122-1)
Supplement: Additional file 1: — Supplemental Figures S1-S5, supplemental Table S1 and supplemental Methods. [file 13041_2015_122_MOESM1_ESM.pdf]

A

```
C_el_Smg4     NFSEYCDSMMEFERRFDGYIFVDSRGNDSAAVVEAASNQNFAKCDRNRMKEDTRVGAILTDKYYLDFCKKLEEERAIPILTLEQQIRKLNQPDDARTQIDKMETPLVKY
H_sa_UPF3A    NFRN-PDDILLFRDRFDGYIFLDSK-----------------------------------DPEYKKFLETYCVEEEKTSANPETLLGEMEAKT--RELIARRTTPLLEY
D_re_UPF3B    NFKN-QDDIVLFRDRFDGYVFIDNRGQEYPAIVEFAPFQKVAK--KRSKKKDAKSGTIDDDADYKKFLEFYNGDEEKSPSNPEILLEEIEAKT--KELSSKKTTPLLDF
X_tr_UPF3B    NFKS-QDDIVLFRDRFDGYVFIDHRGQEYPAIVEFAPFQKVAK--KKSKKKDSKIGTIEDDPEYKKFLDSYTLDEEKLTSTPETLLEEIEAKN--KEMIAKKTTPLLSF
H_sa_UPF3B    NFKN-QEDIILFRDRFDGYVFLDNKGQEYPAIVEFAPFQKAAK--KKTKKRDTKVGTIDDDPEYRKFLESYATDNEKMTSTPETLLEEIEAKN--RELIAKKTTPLLSF
G_ga_UPF3B    NFRN-QEDIVLFRDRFDGYVFVDHKGQEYAAIVEFAPFQKAAK--KKSKKKDAKTGTIEDDPEYKKFLESYSADDEKLTSTPETLLEEIEARN--KELIAKKTTPLLNF
              ** .  :.:: *  *****:*:* .                                   *  * .* .    :      . *  : :::     .    .  ***:.:
                                                                            Y160
```

B

```
R_no      ------------------------------------------------------------  0
H_sa      ------------------------------------------------------------  0
E_pr      MDIQKNAENTTLQNNKRLYRNICITTTQVTKYKTDNTQEATHVPSP-DYNGPPTDTAAFR  59
S_su      ------------------------------------------------------------  0
F_ca      ------------------------------------------------------------  0
O_an      ME---------------------ERVEASPRFSSSSSS-SSSSPTPRRCPGMPRPLCILG  38
G_ga      ------------------------------------------------------------  0

R_no      ------MKEEKDHRPKEKRVTLL-------------TPQGATGGCIGATAEGAKGDDRQD  41
H_sa      ------MKEEKEHRPKEKRVTLL-------------TPAGATGSGGGTSGDSSKGEDKQD  41
E_pr      PSCHCEEREEKEHRPKEKRVTLL-------------TPPGATGSGGGASGDSSKGEDKQD  106
S_su      ------MKEEKDHRPKEKRVTLL-------------TPPGATGSGGGASGDSTKGEDKQD  41
F_ca      ------MKEEKDHRPKEKRVTLL-------------TPPGATGSSGGASGDSAKGEDKQD  41
O_an      LVVHQELPDRASRAKPGRRLQLAAFKRRRSGREAESRGCAFKGGSREMWRKGISC-----  93
G_ga      ------MKEDKENARPKER---------RGAPVGPGAGSGAAGGSDGRTGGGAAELERLE  45
                  :  ..    .*                    .  *.       .

R_no      RNRDKKEALSKVVIRRLPPTLTKEQLQEHLQPMPEHDYFEFFSNDTSLYPHMYARAYINF  101
H_sa      RNKEKKEALSKVVIRRLPPTLTKEQLQEHLQPMPEHDYFEFFSNDTSLYPHMYARAYINF  101
E_pr      RNKEKKEALSKVVIRRLPPTLTKEQLQEHLQPMPEHDYFEFFSNDTSLYPHMYARAYINF  166
S_su      RNKEKKEALSKVVIRRLPPTLTKEQLQEHLQPMPEHDYFEFFSNDTSLYPHMYARAYINF  101
F_ca      RNKEKKEALSKVVIRRLPPTLTKEQLQEHLQPMPEHDYFEFFSNDTSLYPHMYARAYINF  101
O_an      --LFIFWFIFKVVIRRLPPSLTKEQLEEHLQPLPEHDYFEFFANDSSLYPHMFSRAYINF  151
G_ga      RPKDKKETLSKVVIRRLPPSLTREQLEEHLQPLPEHDYFEFFANDSSLYPHVFSRAYINF  105
                  : *********:**:***:*****:*********:**:*****:::******
```

```
R_no      KNQEDILLFRDRFDGYVFLDNKGQEYHAIVEFAPFQKAAKKKIKKRDTKVGTIEDDPEYR  161
H_sa      KNQEDIILFRDRFDGYVFLDNKGQEYPAIVEFAPFQKAAKKKTKKRDTKVGTIDDDPEYR  161
E_pr      KNQEDIILFRDRFDGYVFLDNKGQEYPAIVEFAPFQKAAKKKTKKRDTKVGTIDDDPEYR  226
S_su      KNQEDIILFRDRFDGYVFLDNKGQEYPAIVEFAPFQKAAKKKTKKRDTKVGTIDDDPEYR  161
F_ca      KNQEDIILFRDRFDGYVFLDNKGQEYPAIVEFAPFQKAAKKKTKKRDTKVGTIDDDPEYR  161
O_an      KNQEDIVLFRDRFDGYVFIDHKGQEYPAIVEFAPFQKSAKKKSKKKDAKTGTIDEDPEYK  211
G_ga      RNQEDIVLFRDRFDGYVFVDHKGQEYAAIVEFAPFQKAAKKKSKKKDAKTGTIEDDPEYK  165
          :*****:***********:*.***** **********:**** **:*:*.***::****:
                                                                Y160

R_no      KFLESYATDNEKMTSTPETLLEEIEAKNRELIAKRTTPLLSFLKNKQRMREEKREERRRR  221
H_sa      KFLESYATDNEKMTSTPETLLEEIEAKNRELIAKKTTPLLSFLKNKQRMREEKREERRRR  221
E_pr      KFLESYAADNEKMTSTPETLLEEIEAKNRELIAKKTTPLLSFLKNKQRMREEKREERRRR  286
S_su      KFLESYAADNEKMTSTPETLLEEIEAKNRELIAKKTTPLLSFLKNKQRMREEKREERRRR  221
F_ca      KFLESYAADNEKMTSTPETLLEEIEAKNRELIAKKTTPLLSFLKNKQRMREEKREERRRR  221
O_an      KFLESYSADDEKLTSTPETLLEEIEARNKELIAKKTTPLLNFLKNKQRLREEKREERRRR  271
G_ga      KFLESYSADDEKLTSTPETLLEEIEARNKELIAKKTTPLLNFLKNKQRLREEKREERRRR  225
          ******::*:**:*************:*:*****:*****.*******:***********

R_no      EIERKRQREEEERRKWKEEEKRKRKDIEKLKKIERIPERE--KIRDEPKIK----------  269
H_sa      EIERKRQREEEERRKWKEEEKRKRKDIEKLKKIDRIPERD--KLKDEPKIK----------  269
E_pr      EIERKRQREEEERRKWKEEEKRKRKDIEKLKKIDRVPERD--KLKDEPKIKVHRFLLQAVN  344
S_su      EIERKRQREEEERRKWKEEEKRKRKDIEKLKKIDRVPERD--KLKDEPKIK----------  269
F_ca      EIERKRQREEEERRKWKEEEKRKRKDIEKLKKIDRVPERD--KLKDEPKIK----------  269
O_an      EIERKRQREEEERRKWKEEERRKRKDAEKLKKLERGPEKERDRSKDEPKIK----------  321
G_ga      ELERKRQREEEERRKWKEEERRKRKEAEKLKKVDRCPEKERDRSKEEPKIK----------  275
          *:******************:****: *****::* **::  : ::*****
                                         R255

R_no      ---LLRKPEKGDEKELDKRDKTKRLDKENLNEDRASGHSYTLPRRSDVELKDEKPKRPDD  326
H_sa      ---LLKKPEKGDEKELDKREKAKKLDKENLSDERASGQSCTLPKRSDSELKDEKPKRPED  326
E_pr      QKNLLKKPEKGDEKELDKREKAKKLDKENLNDERASGQSCTLPKRSDGELKEEKPRRPED  404
S_su      ---LLKKPEKGDEKELDKREKVKKLDKENLSDERASGQTCTLPKRPDGEFKDEKPKRPED  326
F_ca      ---LLKKPEKGDEKELDKREKAKKLDKENLNDERASGQSCTLPKRSDGEPKDEKPKRPED  326
O_an      ---LLKKPEKGDEMESEKREKPKRLDKENLNEEKSSGQSSTSAKRSDGEAKEDKAKKSED  378
G_ga      ---LLKKPEKDEK-DLEKKEKSKKLEKETLREEKN--ASSASAKRSDGETKEEKAKKSED  329
             **:**** :: : :*::* *:*:**.* :::    : :  :* * * *::* :: :*
```

```
R_no      ESVRDYRDR--DRDYERDQERMIRERERMKRQEEERRRQQQKERYEKEKAFKRKEEEMKR  384
H_sa      ESGRDYRER--EREYERDQERILRERERLKRQEEERRR--QKERYEKEKTFKRKEEEMKK  382
E_pr      ESGRDYRER--DRDYERDQERILRERERLKRQEEERRR--QKERYEKEKAFKRKEEEVKK  460
S_su      ESSRDYRER--ERDYERDQERILRERERLKRQEEERRR--QKERYEKEKAFKRKEEEMKK  382
F_ca      EGGRDYREREREREDYERDQERLLRERERLKRQEEERRR--QKERYEKEKAFKRKEEEMKK  384
O_an      EGGKDYRERDKDFERDRDRERVQRDREKMRRQEEERRR--QRERVEKERVFRRKEEDVRK  436
G_ga      ECVKDYRDRDRDFERDREYERAQ--REKLRRQEEERRR--QKERFEKEKVFRRKEEEMKK  385
          *  :***:*  : : :*: **    **:::********  *:** ***:.*:****::::
                                      R368         R379

R_no      EKEALRDKGKKSENTESICSLEKI------EKKEEVVKRDRIRNKDRPAMQLYQPGARSR  438
H_sa      EKDTLRDKGKKAESTESIGSSEKT------EKKEEVVKRDRIRNKDRPAMQLYQPGARSR  436
E_pr      EKEALRDKGKKTESTESVGSSEKT------EKKEEVVKRDRIRNKDRPAMQLYQPGARSR  514
S_su      EKEALRDKAKKTESTEPVGSSEKT------EKKEEVVKRDRIRNKDRPAMQLYQPGARSR  436
F_ca      EKEAVRDKGKKPESTESVGSSEKT------EKKEEVVKRDRIRNKDRPAMQLYQPGARSR  438
O_an      ERDPLRDKVKKSESTDFIGNSEKTDKVTKEDKKEDAAKRDRIRNKDRPAMQLYQPGARSR  496
G_ga      ERDLLRDKGKKSDLTDFTCGMDKSEKVTKDDKKEDTVKRDRIRNKDRPAMQLYQPGARSR  445
          *:: :*** ** : *:     :*        :***:..***********************

R_no      SRLCPADDSI-KPGDSPVEKKQESGISHRKEGGEE   472
H_sa      NRLCPPDDST-KSGDSAAERKQESGISHRKEGGEE   470
E_pr      NRLCPPDDST-KSGDSAIEKKQESGISHRKEGEE-   547
S_su      NRLCPPDDST-KSGDSTIDKKQESGISHRKEGGEE   470
F_ca      NRLCPPDDGT-KSGDAALEKKQESGISHRKEGGEE   472
O_an      SRLGPYEDSSAKSADPAPDKKQECEITSRKEEEE-   530
G_ga      SRLCQYEDSAAKSTDQGAEKKQESESSNMKEEE--   478
          .**   :*.  *  *   ::***.  :  **
```

Fig. S1. A. Alignment of animal UPF3 proteins including human UPF3A and UPF3B. The region flanking the conserved Y160 residue is shown. B. Alignment of mammalian and chicken UPF3B proteins. The conserved N-terminal RNP domain and C-terminal EJC interaction domain are on gray background [1,2]. The residues changed in patients with neurodevelopmental disorders are highlighted. The alignment was done using Clustal Omega [3]. Abbtreviations/protein identifiears are: C_el: *C. elegans* [GenBank:NP_741600]; D_re: *Danio rerio* [GenBank:NP_957248]; E_ca: *Equus przewalskii* [GenBank:XP_008507162]; F_ca: *Felis catus* [GenBank:XP_004000884]; G_ga: *Gallus gallus* [GenBank:XP_003641170]; H_sa: *Homo sapiens [UPF3A:* GenBank:NP_542418; UPF3B: GenBank:NP_957248] ; M_mu: *Mus musculus* [GenBank:NP_080849]; O_an: *Ornithorhynchus anatinus* [GenBank:XP_001509880]; R_no: Rattus norvegicus [GenBank:NP_001129345]; S_cr: *Sus scrofa* [GenBank:XP_003135388]; X_tr: *Xenopus tropicalis* [GenBank:XP_002940330].

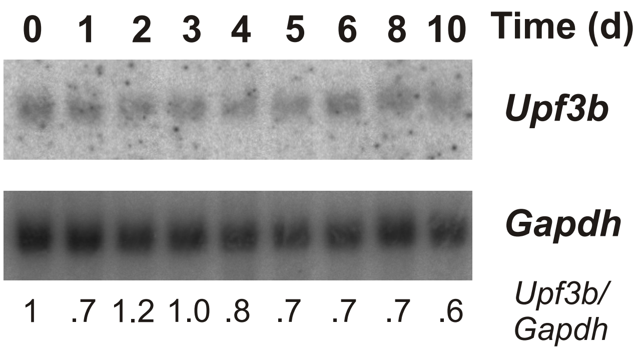

Fig. S2. Upf3b mRNA levels decrease during neuronal differentiation of neural stem cells. Neural stem cells were induced to differentiate and total mRNA was prepared before (day 0 (d 0)) and after beginning of differentiation (days 1 - 10 (d 1 - d 10)). *Upf3b* and *Gapdh* mRNA levels were analysed by Northern blotting as described (Zhao et al., 2004). Relative *Upf3b* mRNA levels were standardised with respect to *Gapdh* mRNA levels, with levels at day 0 defined as 1.

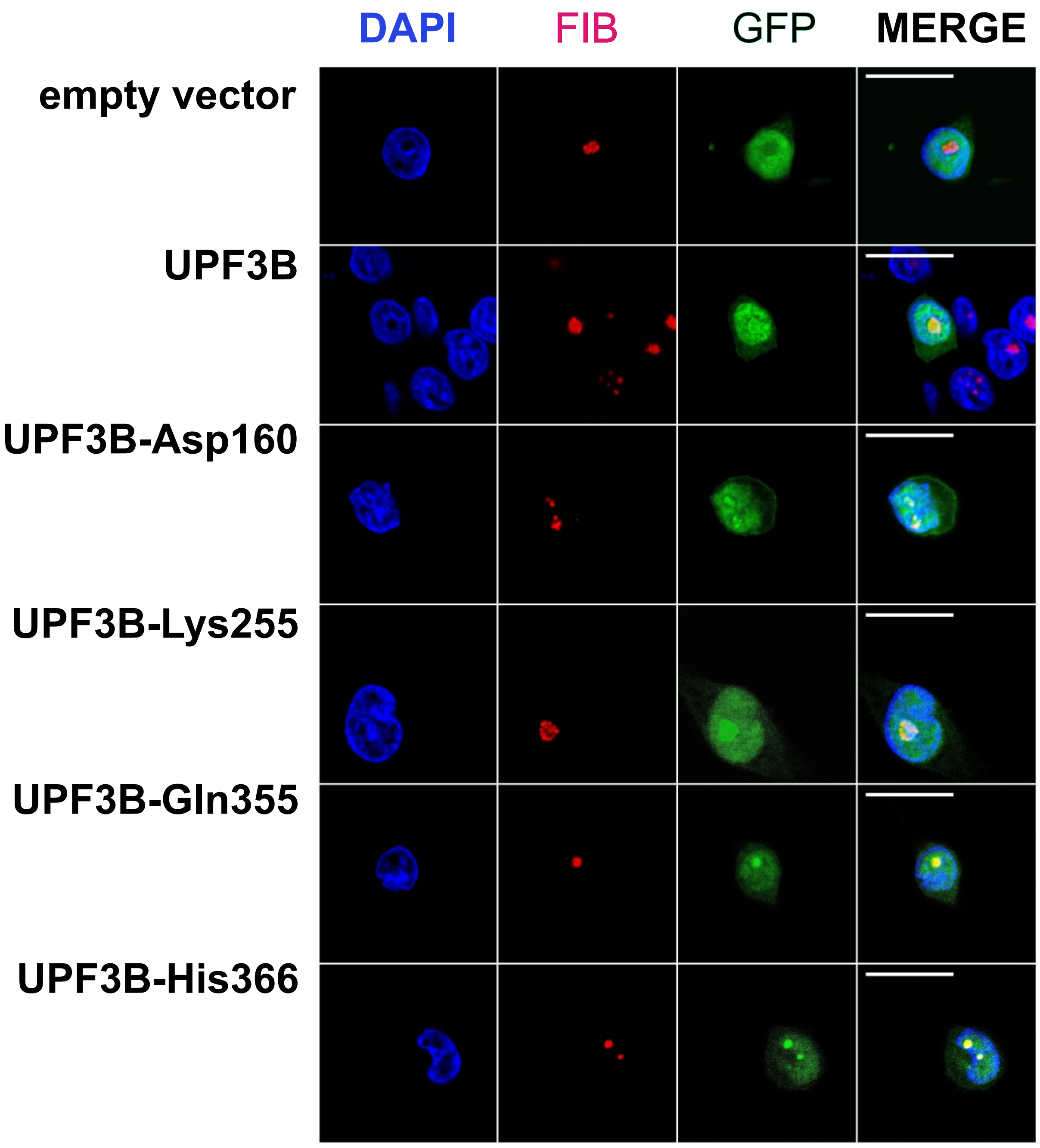

Fig. S3. Nuclear localisation and nucleolar enrichment of UPF3B proteins. Neural stem cells were transfected with the pEGFP-C3 expressing GFP only (empty vector) or pEGFP-C3 derivatives expressing one of the UPF3B forms with an N-terminal GFP tag. Cells were diluted and re-plated after 24 hours, and then differentiated for 6 days prior to fixation and staining with anti-Fibrillarin antibody. Cells were analysed by confocal microscopy and shown are single sections focusing on nuclei stained with DAPI, anti-Fibrillarin antibody (Fib) and detecting GFP, respectively, as well as merged pictures. Note the enrichment of GFP-tagged UPF3B proteins, but not of GFP, in nucleoli. The scale bar represents 20 μm.

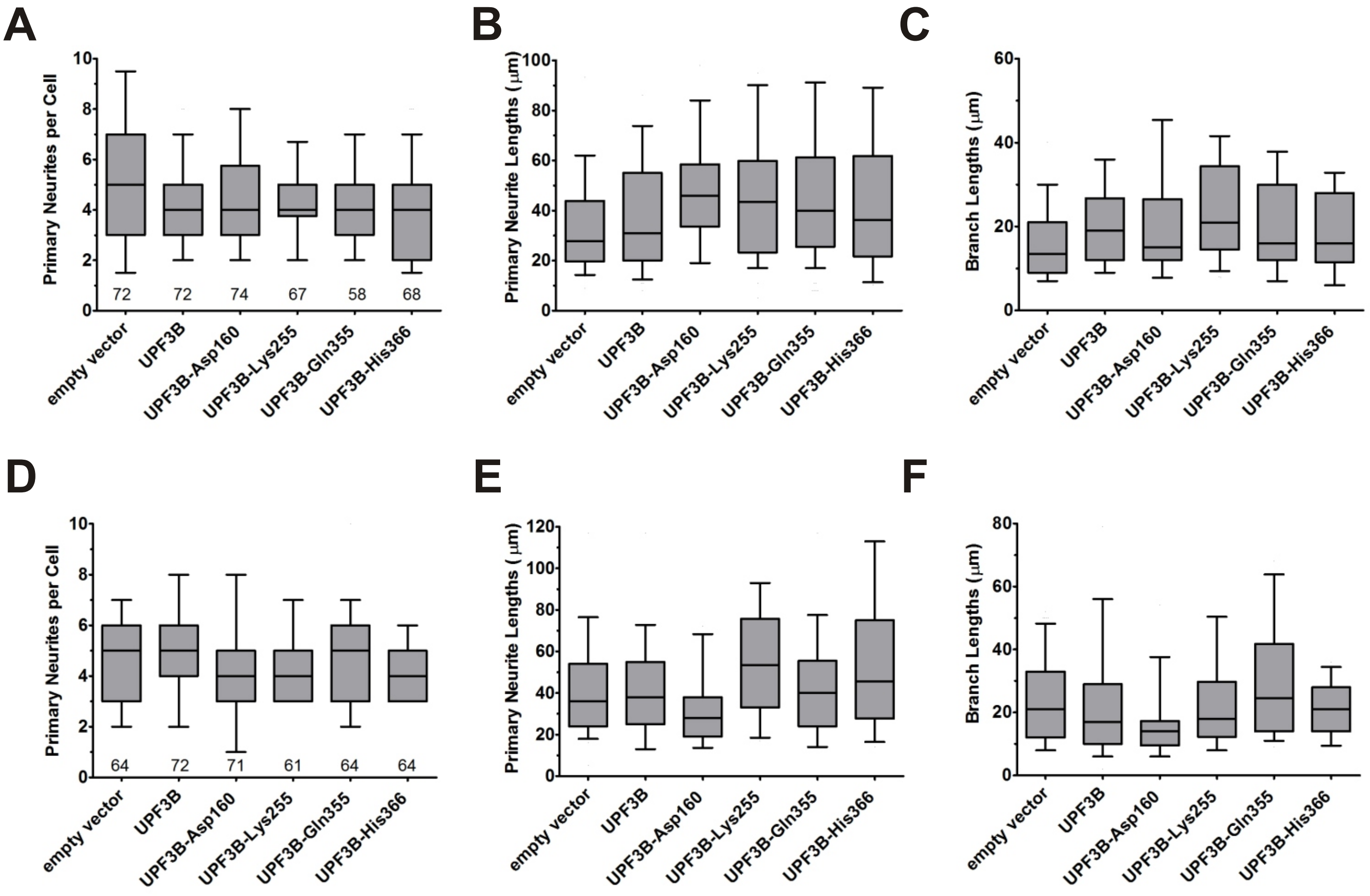

Fig. S4. Effect of mutant UPF3B proteins on neuronal differentiation of neural stem cells. Neurons were transfected and analysed as described for Figure 5. A-F. Analysis of the number of primary neurites per cell and of the distribution of lengths of primary neurites and branches after 3 days (top) and 6 days (bottom) of differentiation. Box plots show the median, with boxes spanning 25th- 75th percentiles and the crossbars indicating the 10th and 90th percentiles. Differences between proteins are not statistically significant (Kruskal Wallis test followed by Dunn's multiple comparison test; *P*<0.05.).

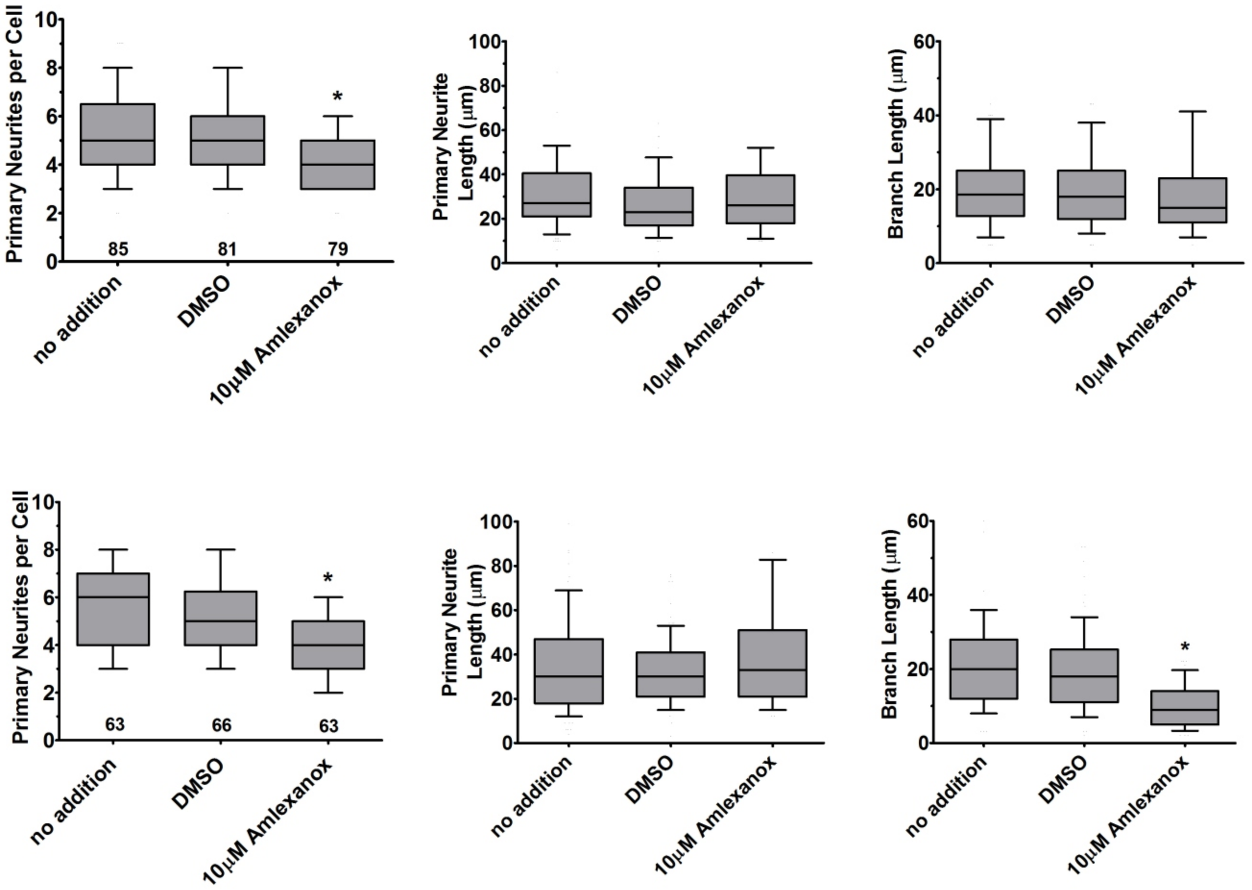

Fig. S5. Effect of Amlexanox treatment on neuronal differentiation. Amlexanox-treated neurons differentiated for 3 days (top) and 6 days (bottom) were analysed as described for Figure 6A. The analysis of primary neurites per cell, primary neurite and branch length per cell are shown. The Asterisk indicates values significantly different from those obtained with cells differentiated without addition of DMSO or Amlexanox (Kruskal Wallis test followed by Dunn's multiple comparison test; *P*<0.05.).

**Table S1: qPCR Assays**		
Target	Primer 1	Primer 2
GFP	AGGACTTCCCCGAGTACCAC	CGTCCTCCACGTAGGTCTTC
Renilla luciferase	CGAGAACGCCGTGATTTT	GACGTGCCTCCACAGGTAG
Gapdh	AGCTGGTCATCAATGGGAAA	ATTTGATGTTAGCGGGATCG
Arhgap24 isoform 1	TCCAGGAAAGTTCCTTTTCG	ATTGGCTGTCATCCGATCTC
Arhgap24	CGAAGACTTTTTGTCCTGTGC	TGTTTCGTTAGCTCCTTCACG
Atf4	TCAGACACCGGCAAGGAG	GTGGCCAAAAGCTCATCTG

Supplemental Methods

Nuclear localisation of UPF3B. HCN-A94 cells were seeded in 6-well plates and the following day were transfected with 5 ug of pEGFP-C3 or derivatives expressing GFP-UPF3B fusion proteins using Lipofectamine 2000. The following day cells were diluted in 35 mm µ-Dishes (Ibidi) and differentiated for six days. Cells were then fixed in 4% paraformaldehyde in PBS, washed three times with PBS and permeabilized in 0.1% Triton X-100. Following 1 hour blocking in 5% normal goat serum (Sigma) in PBS with 0.3% Triton X-100 cells were incubated with mouse anti-Fibrillarin antibody (1:500; Abcam) overnight at 4 ºC in 1x PBS, 1% BSA, 0.3% Triton X-100. The next day cells were washed three times with PBS and incubated with goat anti-mouse Alexa Fluor 594 (Invitrogen) in 1x PBS, 1% BSA, 0.3% Triton X-100. After 1 hour, cells were then washed three times with PBS, blocked for 1 hour and incubated with rabbit anti-UPF3B (1:400; Abgent) in in 1x PBS, 1% BSA, 0.3% Triton X-100 overnight at 4 ºC. Then cells were washed three times with PBS and incubated with goat anti-rabbit Alexa Fluor 888 (1:400, Invitrogen). After 1 hour cells were washed three times with PBS, counterstained with DAPI and washed again three times with PBS. Images were captured using an inverted confocal microscope (Zeiss LSM 710) with 63x oil objective and analysed using ZEN (Zeiss) and ImageJ software [4].

References

1. Kadlec J, Izaurralde E, Cusack S: The structural basis for the interaction between nonsense-mediated mRNA decay factors UPF2 and UPF3. *Nat Struct Mol Biol* 2004, 11: 330-337.

2. Gehring NH, Neu-Yilik G, Schell T, Hentze MW, Kulozik AE: Y14 and hUpf3b form an NMD-activating complex. *Mol Cell* 2003, 11: 939-949.

3. Sievers F, Wilm A, Dineen D, Gibson TJ, Karplus K, Li W *et al*.: Fast, scalable generation of high-quality protein multiple sequence alignments using Clustal Omega. *Mol Syst Biol* 2011, 7: 539.

4. Schneider CA, Rasband WS, Eliceiri KW: NIH Image to ImageJ: 25 years of image analysis. *Nat Meth* 2012, 9: 671-675.
